# Multicentric Longitudinal Prospective Study in a European Cohort of *MYO7A* Patients: Disease Course and Implications for Gene Therapy

**DOI:** 10.1167/iovs.65.6.25

**Published:** 2024-06-17

**Authors:** Francesco Testa, Ester Carreño, L. Ingeborgh van den Born, Paolo Melillo, Irene Perea-Romero, Valentina Di Iorio, Giulia Risca, Clemente Maria Iodice, Ronald J. E. Pennings, Marianthi Karali, Sandro Banfi, Alberto Auricchio, Stefania Galimberti, Carmen Ayuso, Francesca Simonelli

**Affiliations:** 1 Eye Clinic, Multidisciplinary Department of Medical, Surgical and Dental Sciences, University of Campania “Luigi Vanvitelli,” Naples, Italy; 2 Department of Ophthalmology, Health Research Institute-Fundación Jiménez Díaz University Hospital, Universidad Autónoma de Madrid (IIS-FJD, UAM), Madrid, Spain; 3 The Rotterdam Eye Hospital, Rotterdam, the Netherlands; 4 Department of Genetics, Health Research Institute-Fundación Jiménez Díaz University Hospital, Universidad Autónoma de Madrid (IIS-FJD, UAM), Madrid, Spain; 5 Center for Biomedical Network Research on Rare Diseases (CIBERER), Instituto de Salud Carlos III, Madrid, Spain; 6 Bicocca Bioinformatics, Biostatistics and Bioimaging Centre, Department of Medicine and Surgery, University of Milano-Bicocca, Milan, Italy; 7 Department of Otorhinolaryngology, Hearing & Genes, Radboud University Medical Center, Nijmegen, the Netherlands; 8 Medical Genetics, Department of Precision Medicine, University of Campania “Luigi Vanvitelli,” Naples, Italy; 9 Telethon Institute of Genetics and Medicine (TIGEM), Pozzuoli, Italy; 10 Medical Genetics, Department of Advanced Biomedical Sciences, Federico II University, Naples, Italy; 11 AAVantgarde Bio, Milan, Italy

**Keywords:** Usher syndrome, *MYO7A*, natural history study, retinitis pigmentosa

## Abstract

**Purpose:**

We investigated the natural history of retinal dystrophy owing to variants in the *MYO7A* gene.

**Methods:**

Fifty-three patients (mean age, 33.6 ± 16.7 years) with Usher syndrome owing to biallelic, mostly pathogenic, variants in *MYO7A* underwent baseline and two annual follow-up visits. Best-corrected visual acuity (BCVA), semiautomatic kinetic visual field, full-field electroretinogram, color fundus imaging, microperimetry, spectral-domain optical coherence tomography, and fundus autofluorescence were assessed.

**Results:**

At baseline, all patients presented with decreased BCVA (66.4 ± 17.9 Early Treatment Diabetic Retinopathy score and 59.5 ± 21.7 Early Treatment Diabetic Retinopathy score, in the better- and worse-seeing eyes, respectively), restricted semiautomatic kinetic visual field (III4e area, 3365.8 ± 4142.1^°2^; 4176.4 ± 4400.3^°2^) and decreased macular sensitivity (9.7 ± 9.9 dB; 9.0 ± 10.2 dB). Spectral-domain optical coherence tomography revealed reduced central macular thickness (259.6 ± 63.0 µm; 250.7 ± 63.3 µm) and narrowed ellipsoid zone band width (2807.5 ± 2374.6 µm; 2615.5 ± 2370.4 µm). Longitudinal analyses (50 patients) showed a significant decrease of BCVA in better-seeing eyes, whereas no changes were observed in worse-seeing eyes for any parameter. BCVA, semiautomatic kinetic visual field (III4e and V4e) and macular sensitivity were related significantly to age at baseline. Hyperautofluorescent foveal patch (16 eyes [31.4%]) and abnormal central hypoautofluorescence (9 eyes [17.6%]) were significantly associated with worse morphological and functional read-outs compared with the hyperautofluorescent ring pattern (22 eyes [43.1%]).

**Conclusions:**

Our European multicentric study offers the first prospective longitudinal analysis in one of the largest cohorts of *MYO7A* patients described to date, confirming the slow disease progression. More important, this study emphasizes the key role of fundus autofluorescence patterns in retinal impairment staging and advocates its adoption as an objective biomarker in patient selection for future gene therapy clinical trials.

Usher syndrome (USH) is an autosomal recessive disease with an estimated prevalence of 3 to 6 in 100,000 individuals. It is responsible for approximately 50% of hereditary cases of combined deaf–blindness and represents the most common form of syndromic retinitis pigmentosa (RP).^1^^–^^3^ Three USH clinical subtypes and one rare form have been described in literature, and they differ in terms of onset and progression of the sensorineural hearing loss, vestibular dysfunction, and RP severity.^4^

USH type 1 (USH1) usually manifests with a severe to profound congenital sensorineural hearing loss, vestibular areflexia, and early-onset RP.^4^ USH type 2 features moderate to severe congenital sensorineural hearing loss, generally preserved vestibular function, and RP onset in the second decade of life.^4^ Finally, USH type 3 presents a less consistent phenotype, with progressive hearing loss, vestibular dysfunction, and variable age of RP onset.^5^ Recently, a fourth, rarer clinical subtype (USH type 4) has been defined as well.^6^^,^^7^

Besides the variable clinical presentation, USH is characterized by genetic heterogeneity with at least 11 disease-causing genes associated with the three main clinical subtypes, as described in the Online Mendelian Inheritance in Man (OMIM) database.^6^^,^^8^

USH1 accounts for approximately 40% of all USH cases and has been associated with variants in the genes *CDH23*, *CIB2*, *MYO7A*, *PCDH15*, *USH1C*, and *USH1G*.^6^^,^^8^^,^^9^ Biallelic variants in *MYO7A* are responsible for the USH subtype 1B (USH1B; OMIM #276900), which is the most common form of USH1,^11^ and are only exceptionally associated with USH type 2.^10^ These variants affect the normal levels of functional myosin VIIA, causing disorganized sensory hair cells and mechanosensitive hair bundles in the inner ear, and dysfunctional photoreceptors and RPE cells in the retina, perturbing the transduction of mechanoelectrical impulses in both tissues.^12^ In the retina, myosin VIIA deficiency was also reported to impair calyceal processes and outer segment disc morphogenesis in photoreceptor cells, in addition to other abnormalities, such as melanosome and phagosome transport defects in RPE cells and delayed rhodopsin transport in photoreceptors.^12^^,^^13^ Despite our improved understanding of the defects that underpin progressive vision loss in USH1B in animal models,^12^ there remains a substantial need to identify proper disease-predicting variables, biomarkers, and robust genotype–phenotype correlations. Moreover, in the era of emerging retinal therapies, gene augmentation strategies for *MYO7A* retinal degeneration have proved to be successful in treating animal models.^12^^,^^14^ To this point, an accurate phenotyping of such a heterogeneous disorder is warranted to guide disease prognosis and to design clinical trials with relevant inclusion criteria, timing of intervention, and properly chosen therapeutic outcome measurements.

Therefore, the aim of this multicentric natural history study designed by the Center for Rare Ocular Diseases of the University of Campania “Luigi Vanvitelli” (Naples, Italy) was to improve our understanding of the natural history of the disease by (1) gathering data on reproducible and reliable disease patterns, (2) carrying out a quantitative analysis of the clinical parameters, and (3) seeking genotype–phenotype correlations in a large cohort of patients with *MYO7A-*related RP.

## Methods

This study was conducted in compliance with the tenets of the Declaration of Helsinki and subsequent revisions, and with the authorization of the Ethics Committees of the Coordinating Center (University of Campania “Luigi Vanvitelli”, Naples, Italy) and the participating centers (Instituto de Investigacion Sanitaria Fundación Jimenez Diaz, Madrid, Spain; Rotterdam Eye Hospital, Rotterdam, the Netherlands). All patients or their legal guardians gave informed consent before participating in any study procedures. The study was designed as a multicentric, prospective, longitudinal, observational assessment (ClinicalTrials.gov NCT03814499). The primary objective was to evaluate the natural progression of the disease over time in *MYO7A* patients using best-corrected visual acuity (BCVA) and visual field testing.

The inclusion criteria were (a) USH diagnosis, (b) molecular diagnosis of USH owing to *MYO7A* biallelic pathogenic variants, (c) ≥8 years of age at baseline, (d) BCVA of ≥20/640 in at least one eye, and (e) a willingness to adhere to the study protocol for long-term follow-up as evidenced by written informed consent or parental permission and patient assent.

The exclusion criteria were the following: (a) unable or unwilling to meet the requirements of the study, (b) unable to communicate with suitable verbal/auditory and/or tactile sign language (according to the investigator); (c) participation in a clinical study with an investigational drug in the past 6 months, (d) preexisting ocular conditions unrelated to the RP/USH diagnosis that could interfere with the interpretation of the study endpoints in both eyes, such as glaucoma, corneal or significant lenticular opacities, moderate or severe cystoid macular lesions (CMLs),^15^ or macular hole, (e) complicating systemic diseases in which the disease itself, or the treatment for the disease, could alter ocular function (e.g., diabetes, multiple sclerosis etc), (f) prior ocular surgery within the past three months, and (g) any other condition that would hinder the patient from properly completing the follow-up examinations or would render the patient unsuitable for the study.

In case of patients with a BCVA of <20/640 in one eye only (see inclusion criterion e) and preexisting ocular conditions that could interfere with the interpretation of the study endpoints in one eye only (see exclusion criterion d), the contralateral eye was included.

The collected data included demographics (i.e., age and gender); ocular, medical, and surgical history and concomitant treatments; genetic and clinical diagnosis; BCVA; semiautomatic kinetic visual field (SKVF); full-field ERG; microperimetry; color fundus imaging, spectral-domain optical coherence tomography (SD-OCT); and fundus autofluorescence (FAF) imaging.

Cases were coded and data pseudonymized using the following codification system: N0xx for Naples cases; N1xx for Madrid cases, and N2xx for Rotterdam cases.

The clinical diagnosis of USH was made evaluating speech quality, audiological assessments, and the self-reported childhood onset of hearing impairment, in addition to the typical clinical features of RP and rod–cone dystrophy. The clinical diagnosis was further confirmed by molecular genetic testing performed either by panel-based next-generation sequencing analysis of known USH, inherited retinal disease, and/or deafness genes or by clinical and whole exome sequencing. After variant calling and annotation, only variants with minor allele frequency of <0.01 were retained and prioritized considering the variant type, the predicted protein effect, and in silico pathogenicity scores. The selected variants were validated by Sanger sequencing of the corresponding genomic fragments. Segregation analysis was performed in 61% of the cohort, confirming biallelism.

All ophthalmological tests were performed according to standard operating procedures shared among the three centers. BCVA was assessed in both eyes using a standardized Early Treatment Diabetic Retinopathy (ETDRS) protocol.

Clinical ophthalmic examinations included external ocular inspection to assess the motility of extraocular muscles, the appearance and function of eyelids; slit lamp biomicroscopy of eyelids, lashes, conjunctiva, cornea, lens, iris, and anterior chamber; measurements of IOP using either Goldmann applanation tonometry or a handheld applanation tonometer (e.g., Tonopen); and dilated fundus ophthalmoscopy to assess the retina, macula, and optic nerve head.

SKVF testing was performed using an Octopus 900 Pro perimeter with Eye Suite i4.000 software (Haag-Streit International, Koeniz, Switzerland). The background illumination of the bowl was 31.4 apostilb. Data from the stimuli test sizes V4e, III4e, and I4e were collected and the total seeing area was calculated for each isopter (defined as seeing area minus defined scotoma). Test vectors were presented approximately every 15°, at an angular velocity of 4°/s, and originating approximately 10° outside the age-correlated normal isopter. Scotomas were mapped using an angular velocity of 2°/s, originating from the assumed center using ≥12 vectors. Blind spot mapping was done with the smallest test size target.

Microperimetry, when available, was performed using a fundus-related perimeter (MAIA; CenterVue, Padova, Italy) before retinal examination and imaging. The grid used to assess macular sensitivity (MS) consisted of 68 test loci arranged in a square array centered on the fovea. Adjacent test loci were separated by 2° at 1°, 3°, 5°, 7°, and 9° from the vertical or horizontal meridian. Goldmann III achromatic stimuli with stimulus duration of 200 ms were presented on a dim white background (1.27 cd/m^2^) one at a time. The dynamic range of the stimulus luminance was 0.08 to 317.04 cd/m^2^, corresponding with a range of 36 to 0 dB. Test strategy was a 4-2 staircase.

Full-field ERG was recorded in both eyes, using Reticom (Roland Consult, Brandenburg an der Havel, Germany) or Espion D15 (Diagnosys LLC, Lowell, MA, USA) equipment, in accordance with the standard International Society for Clinical Electrophysiology of Vision guidelines.^16^

Infrared reflectance and FAF (blue) imaging covering either the central 30°or 35°of the posterior pole were obtained using Heidelberg Spectralis (Heidelberg Engineering GmbH, Heidelberg, Germany) or Topcon TR-50DX (Topcon Corporation, Tokyo, Japan), respectively. Macular FAF appearance was classified into three different categories, namely, hyperautofluorescent ring, hyperautofluorescent foveal patch, and abnormal central hypoautofluorescence (atrophy), as previously reported.^17^

SD-OCT (Spectralis, Heidelberg Engineering GmbH, Heidelberg, Germany) volume scans were acquired, capturing 121 b-scans in a 30° (horizonal) × 25° (vertical) area of the posterior pole in a high-resolution mode with automatic real-time tracking of 9. Similarly, a horizontal and vertical 30° high-resolution 1-line b-scans with an automatic real-time tracking of >30 were also taken.

To prevent bias and improve consistency of the grading, all available imaging, that is, SD-OCT scans and FAF imaging, were reviewed by two medical retina experts with experience in inherited retinal dystrophies at the Coordinating Centre (FT and VDI independently assessed, followed by the independent assessment of a third senior grader, FS, to confirm the collected measures or in case of discordant qualitative or quantitative findings). Specifically, the presence of the following features was evaluated from SD-OCT data: CML, macular atrophy, and vitreoretinal interface abnormalities. Furthermore, the ellipsoid zone (EZ) width (both on high-resolution horizontal and vertical scans) was measured independently and manually by two experienced researchers (FT and VDI). The central macular thickness (CMT) was detected automatically by the Heidelberg Engineering proprietary software after visual inspection of the segmentation, followed by manual correction when needed.

### Statistical Analysis

Descriptive statistics, that is, mean ± SD and counts (frequency), were assessed. Comparisons between groups were performed by means of the Fisher exact test. The evaluation of the longitudinal changes in functional and morphological parameters over the annual follow-up visits was done by fitting mean response profile models.^18^ This approach is adopted commonly in the analysis of clinical trials or observational studies when the sample size is limited and the measures of the variables of interest are obtained at fixed prespecified time intervals.^19^^,^^20^ The proper variance–covariance matrix (unstructured) was selected within a set of competing forms as the one that minimized the Akaike information criterion. The models were performed both in the better- and in the worse-seeing eyes, adjusting for age or for other covariates measured at baseline (e.g., lens status and FAF patterns). All the tests were two-sided with α = 5%. The analyses were performed using R (R Foundation for Statistical Computing, Vienna, Austria, version 4.3.1).

## Results

### Clinical Characterization of the *MYO7A* Cohort

A total of 62 Caucasian patients (32 recruited in Italy, 21 in Spain, and 9 in the Netherlands) with a clinical diagnosis of USH were screened for inclusion in the study. Nine patients were excluded either because the genetic analysis did not confirm biallelic variants in the *MYO7A* gene (*n* = 3 patients) or owing to the presence of moderate/severe CML or ungradable OCT in both eyes owing to a vitreoretinal pathology (*n* = 6 patients). Therefore, 53 patients (mean age, 33.6 ± 16.7 years; range, 8–72 years), 30 males (56.6%) and 23 females (43.4%), with a clinical and molecular diagnosis of USH-related RP met all the inclusion/exclusion criteria and were further investigated. Moreover, one eye of two patients (NH016 and NH032) was excluded from the analysis owing to moderate or severe CML, and one eye of four patients was excluded either because BCVA was <20/640 (NH005, NH105, and NH022) or owing to severe CML (NH021). As a result, data from 100 eyes (51 evaluated as better-seeing eyes and 49 as worse-seeing eyes) of 53 patients were analyzed in the current study. None of the included patients participated in any prior investigational drug study.

Table 1 summarizes the main clinical features of the study cohort at baseline in the better- and worse-seeing eyes. The mean baseline BCVA in the better-seeing eyes was 66.4 ± 17.9 ETDRS score (corresponding with 20/50), and slightly lower in the worse-seeing eyes, i.e., 59.5 ± 21.7 ETDRS score (corresponding with 20/80). Thirty-one patients (58.5%) showed a BCVA of ≥20/40 in at least one eye, whereas only 4 patients (7.5%) were legally blind (i.e., BCVA of ≤20/200 in both eyes). Six patients showed the same score in both eyes and in these patients the better-seeing eye was identified on the basis of the better SKVF. The anterior segment biomicroscopy in the better-seeing eyes revealed clear lens in 20 cases (39.2%), and lens opacities in 19 (37%), whereas the remaining 12 (23.5%) were pseudophakic (Table 2). A similar distribution was observed also in the worse-seeing eyes (Table 2). The patients, on average, showed a markedly restricted SKVF in the better-seeing eyes using both the III4e (3365.8 ± 4142.1^°2^) and V4e stimulus sizes (5605.2 ± 5290.2^°2^). Because the I4e stimulus could be seen only by nine patients (17.0%), the relative data were not analyzed. Similar findings were observed in the worse-seeing eyes (see Table 1). Also, microperimetry showed a significantly decreased MS, that is, 9.7 ± 9.9 dB and 9.0 ± 10.2 dB in the better- and worse-seeing eyes, respectively, compared with manufacturer-provided normative reference database. Finally, SD-OCT revealed, on average, a slightly decreased CMT both in the better- (259.6 ± 63.0 µm) and worse-seeing eyes (250.8 ± 65.3 µm) when compared with normative data (280.1 ± 17.5 µm),^21^ whereas the horizontal and vertical widths of the EZ band were markedly decreased in both the better- and worse-seeing eyes (Table 1). Furthermore, SD-OCT scans revealed mild CML in 5 better-seeing eyes and in 11 worse-seeing eyes, whereas macular atrophy was observed in both eyes of 12 patients.

**Table 1.** Main Clinical Features of Patients at Baseline in the Better- and Worse- Seeing Eyes

	Better-Seeing Eyes		Worse-Seeing Eyes	
Variables	Mean	SD	Mean	SD
BCVA (ETDRS score)	66.4	17.9	59.5	21.7
III4e SKVF area (°^2^)	3365.8	4142.1	4176.4	4400.3
V4e SKVF area (°^2^)	5605.2	5290.2	5613.22	5127.2
MS (dB)	9.7	9.9	9.0	10.2
CMT (µm)	259.6	63.0	250.7	63.3
EZ horizontal width (µm)	2807.5	2374.6	2615.5	2370.4
EZ vertical width (µm)	2436.1	2286.4	2332.4	666.5

**Table 2.** Evaluation of Lens Status, Macular Abnormalities, and FAF Patterns in the Cohort of Patients Stratified by Age

	Overall Cohort		Patients Aged 8–20 Years		Patients Aged 21–40 Years		Patients Aged >40 Years		*P* Value	
Variables	Better-Seeing Eyes^*^ (*n* = 51)	Worse-Seeing Eyes^*^ (*n* = 49)	Better-Seeing Eyes^*^ (*n* = 13)	Worse-Seeing Eyes^*^ (*n* = 12)	Better-Seeing Eyes^*^ (*n* = 20)	Worse-Seeing Eyes^*^ (*n* = 21)	Better-Seeing Eyes^*^ (*n* = 18)	Worse-Seeing Eyes^*^ (*n* = 16)	Better-Seeing Eyes	Worse-Seeing Eyes
Lens status										
Clear lens	20 (39.2)	17 (34.7)	11 (84.6)	9 (75.0)	7 (35.0)	6 (28.6)	2 (11.1)	2 (12.5)	<0.001	0.001
Lens opacities	19 (37.3)	18 (36.7)	2 (15.4)	2 (16.7)	11 (55.0)	11 (52.4)	6 (33.3)	5 (31.2)		
Pseudophakia	12 (23.5)	14 (28.6)	0 (0)	1 (8.3)	2 (10.0)	4 (19.0)	10 (55.6)	9 (56.2)		
Mild CMLs	5 (9.8)	11 (22.4)	2 (15.4)	4 (33.3)	1 (5.0)	4 (19.0)	2 (11.1)	3 (18.8)	0.602	0.582
Macular atrophy	12 (23.5)	11 (22.4)	0 (0)	0 (0)	5 (25.0)	5 (23.8)	7 (38.9)	6 (37.5)	0.067	0.085
FAF patterns										
No abnormalities	3 (5.9)	3 (6.1)	1 (7.7)	1 (8.3)	2 (10.0)	2 (9.5)	0 (0.0)	0 (0.0)	0.001	0.009
Hyperautofluorescent ring	22 (43.1)	20 (40.8)	12 (92.3)	10 (83.3)	8 (40.0)	8 (38.1)	2 (11.1)	2 (12.5)		
Patch pattern	16 (31.4)	16 (32.7)	0 (0)	0 (0)	6 (30.0)	7 (33.3)	10 (55.6)	9 (56.2)		
Severely decreased FAF	9 (17.6)	9 (18.4)	0 (0)	1 (8.3)	3 (15.0)	3 (14.3)	6 (33.3)	5 (31.2)		
Central hyperautoflu-orescence	1 (2.0)	1 (2.0)	0 (0	0 (0)	1 (5.0)	1 (4.8)	0 (0	0 (0)		

* Data are expressed as count (frequency).

With regard to FAF evaluation in the best-seeing eyes, the most common pattern was hyperautofluorescent ring (22 cases [43.1%]), followed by hyperautofluorescent foveal patch (16 eyes [31.4%]) and abnormal central hypoautofluorescence (9 eyes [17.6%]). Only one eye (2.0%) displayed central hyperautofluorescence, whereas three eyes (5.9%) had a normal FAF appearance. Similar findings were observed in the worse-seeing eyes (Table 2). Table 2 also provides a comparison of patients stratified according to the age group (8–20 years, 21–40 years, and >40 years), showing a significantly (*P* < 0.01) higher frequency of eyes with clear lens (84.6%) and of eyes with hyperautofluorescent ring (83.3%) in the group of younger patients compared with the other two groups.

Finally, full-field ERG showed undetectable scotopic and photopic responses in 47 patients (88.7%; mean age, 35.8 ± 15.8 years; range, 10–72 years), whereas subnormal photopic responses were detectable in 5 individuals (9.4%; mean age, 13.0 ± 3.8 years; range, 8–18 years) with undetectable scotopic responses. Only one patient (1.9%), aged 31 years, showed both subnormal scotopic and photopic responses.

Longitudinal analysis, computed on data from 50 patients, is summarized in Table 3. In particular, the 2-year prospective follow-up was completed by all patients except three (NH001 and NH110 who missed the second follow-up visit; and NH206 who missed both the first and second follow-up visits). Longitudinal age-adjusted models revealed a statistically significant decrease of BCVA in the better-seeing eyes (first follow-up visit vs. baseline, −2.3 ± 0.5 ETDRS letters [*P* = 0.0001]; second follow-up visit vs. baseline, −3.8 ± 0.9 ETDRS letters [*P* < 0.0001]), whereas no significant changes were observed in the worse-seeing eyes. No significant worsening was observed for the other functional and morphological parameters (i.e., SKVF area, MS, CMT, and EZ band width) both in worse- and better-seeing eyes, even though a trend of reduction of the EZ band horizontal width was evident in better-seeing eyes (first follow-up visit vs. baseline, −47.1 ± 51.6 µm [*P* = 0.3634]; second follow-up visit vs. baseline, −204.7 ± 111.0 µm [*P* = 0.0677]). Furthermore, BCVA, III4e and V4e SKVF area, and MS were significantly (*P* < 0.01) related to age at baseline in both the better- and worse-seeing eyes. Regarding the SD-OCT parameters, only the EZ band horizontal width in the better-seeing eyes was significantly related to age (*P* = 0.0094), whereas this relationship was not statistically significant in the worse-seeing eyes (*P* = 0.098). Moreover, as summarized in Supplementary Table S1, we performed a subgroup analysis by age group (8–20 years, 21–40 years, and >40 years). In particular, the analysis showed that the worsening of BCVA only in the better-seeing eye was significant (*P* < 0.001) in patients >20 years old, whereas younger patients showed a significant decrease (*P* = 0.0017) at the second follow-up visit only in the worse-seeing eye. SKVF data showed a significant decrease (*P* = 0.0071) only of the III4e area in the worsening eye in the patients aged 21 to 40 years. As regards the SD-OCT parameters, we observed a significant (*P* < 0.01) decrease of EZ band width on the horizontal scan in the subgroup of patients aged 21 to 40 years in both better- and worse-seeing eyes, and a significant decrease (*P* = 0.048) of the EZ band width on vertical scan in the patients aged ≥40 years.

**Table 3.** Longitudinal Age-Adjusted Models of the Selected Functional and Morphological Parameters

		Better-Seeing Eyes			Worse-Seeing Eyes		
Parameter	Factor or Variable	Coefficient	SE	*P* Value^*^	Coefficient	SE	*P* Value^*^
BCVA (ETDRS score)	Baseline	78.7	5.1	<0.0001	83.0	6.3	<0.0001
	First year	**−2.3**	**0.5**	**0.0001**	−0.6	0.8	0.4178
	Second year	**−3.8**	**0.9**	**<0.0001**	−1.5	1.3	0.2677
	Age adjustment^†^	**−0.4**	**0.1**	**0.0075**	**−0.7**	**0.2**	**0.0001**
III4e SKVF area (^°2^)	Baseline	7653.8	966.5	<0.0001	7346.0	903.6	<0.0001
	First year	369.9	375.7	0.3269	−467.0	483.8	0.3364
	Second year	365.4	458.0	0.4265	−908.0	567.5	0.1124
	Age adjustment^†^	**−136.5**	**25.1**	**<0.0001**	−105.5	21.9	**<0.0001**
V4e SKVF area (^°2^)	Baseline	12154.6	1265.7	<0.0001	11788.8	1229.6	<0.0001
	First year	264.3	371.6	0.4782	479.2	427.8	0.2649
	Second year	730.2	661.3	0.2716	1114.3	896.0	0.216
	Age adjustment^†^	**−199.3**	**33.6**	**<0.0001**	**−195.5**	**33.2**	**<0.0001**
MS (dB)	Baseline	20.4	3.1	<0.0001	20.3	3.6	<0.0001
	First year	0.1	0.5	0.9032	−0.1	0.6	0.9349
	Second year	−0.4	1.4	0.7889	−0.6	1.6	0.7196
	Age adjustment^†^	**−0.4**	**0.1**	**0.0001**	**−0.4**	**0.1**	**0.0007**
CMT (µm)	Baseline	272.5	19.7	<0.0001	286.2	22.0	<0.0001
	First year	**7.0**	**3.1**	**0.0242**	4.7	2.4	0.0542
	Second year	1.5	2.3	0.5092	2.6	3.2	0.4224
	Age adjustment^†^	−0.4	0.5	0.4675	−1.1	0.6	0.0772
EZ horizontal width (µm)	Baseline	4403.2	711.1	<0.0001	3684.8	737.7	<0.0001
	First year	−47.1	51.6	0.3634	−118.9	76.9	0.1247
	Second year	−204.7	111.0	0.0677	−135.2	92.4	0.1461
	Age adjustment^†^	**−51.2**	**19.4**	**0.0094**	−34.1	20.5	0.098
EZ vertical width (µm)	Baseline	3405.0	698.6	<0.0001	3301.9	746.2	<0.0001
	First year	−235.8	165.2	0.1560	−154.2	84.3	0.0699
	Second year	−102.0	101.1	0.3148	−164.6	96.3	0.0901
	Age adjustment^†^	−29.2	18.9	0.1257	−28.8	20.8	0.1692

Longitudinal analysis shows a significant reduction of BCVA in the better-seeing eyes. BCVA, V4e SKVF area, and MS are strongly age-related either in the better- and worse- seeing eyes. III4e SKVF area and EZ horizontal width are strongly age-related in the analysis including better-seeing eyes.

* *P* values are obtained by linear mixed models; statistically significant *P* values are shown in bold.

† The models are adjusted for the age of the patients at the baseline visit.

In the better-seeing eyes, lens opacities showed a significant decrease in III4e (*P* = 0.018) and in V4e SKVF area (*P* = 0.01) with respect to clear lenses, whereas the decrease in BCVA and MS was not statistically significant (*P* = 0.252 and *P* = 0.083, respectively). Similarly, pseudophakia was associated with significantly decreased III4e and V4e SKVF areas (*P* < 0.001) and lower MS (*P* = 0.0229) with respect to clear lens, whereas the decrease in BCVA was not statistically significant (*P* = 0.0722). However, these decreases were not statistically (*P* > 0.05) significant after age adjustment, suggesting that the worsening was mainly related to age and not to the development of lens opacities or cataract surgery.

Macular atrophy was significantly associated with reduced BCVA (*P* < 0.001), V4e SKVF area (*P* = 0.0151), MS (*P* = 0.0056), CMT (*P* = 0.0005), and EZ band width on horizontal (*P* = 0.0001) and vertical scans (*P* = 0.0008)

By comparing the most frequent FAF patterns (i.e., hyperautofluorescent ring, hyperautofluorescent foveal patch, and abnormal central hypoautofluorescence), we observed that the hyperautofluorescent ring was associated with a significantly (*P* < 0.0001) younger age (22.5 ±13.4 years) compared with hyperautofluorescent foveal patch (45.7 ± 16.3 years) and abnormal central hypoautofluorescence (41.9 ± 5.6 years). Furthermore, in better-seeing eyes, a hyperautofluorescent foveal patch pattern and abnormal central hypoautofluorescence were associated with significantly (*P* < 0.05) worse BCVA, III4e and V4e SKVF area, and MS compared with a hyperautofluorescent ring, with greater differences observed between abnormal central hypoautofluorescence and hyperautofluorescent ring (Table 4). In particular, the mean BCVA was 77.6 ± 5.2 in eyes with hyperautofluorescent ring, 61.5 ± 17.1 in those with hyperautofluorescent foveal patch pattern, and 44.2 ± 16.9 in those with abnormal central hypoautofluorescence. On average, SKVF was more constricted in eyes with hyperautofluorescent foveal patch pattern (III4e: 2703.6 ± 4267.9^°2^; V4e: 4298 ± 5218.7^°2^) and in those with abnormal central hypoautofluorescence (III4e, 1095.0 ± 1062.4^°2^; V4e, 2775.9 ± 1614.1^°2^) compared with those with hyperautofluorescent ring (III4e: 4669.4 ± 4594.9^°2^; V4e: 7196.7 ± 5610.5^°2^). Finally, MS was more reduced in eyes with a hyperautofluorescent foveal patch pattern (4.2 ± 5.5 dB) and in those with abnormal central hypoautofluorescence (0.4 ± 0.6 dB) compared with those with a hyperautofluorescent ring (13.6 ± 8.1 dB). Moreover, CMT and EZ band width were significantly (*P* < 0.001) decreased in the eyes showing a hyperautofluorescent foveal patch pattern and in those with abnormal central hypoautofluorescence, with lower values associated with the latter FAF appearance. Figure shows the comparison of SD-OCT and SKVF in three selected eyes with different FAF patterns. Similar findings were observed in the worse-seeing eyes, even if the differences in the III4e SKVF area and EZ band vertical width did not attain statistical significance (*P* > 0.05). Finally, over the 2-year follow-up, we observed no changes in the FAF patterns of any patient, except two cases (NH007, aged 50 years, p.[(Ala26Glu)]; [(Arg241Gly)] and NH025, aged 23 years, p.[(Arg669*)];[(Gly1298Arg)]) who progressed from an hyperautofluorescent foveal patch pattern at baseline to abnormal central hypoautofluorescence at the last follow-up visit in both eyes.

**Table 4.** Comparison of the Functional and Morphological Parameters in the Patients Stratified According to the Autofluorescence Patterns

		Better-Seeing Eyes			Worse-Seeing Eyes		
Parameter	FAF Pattern	Coefficient	SE	*P* Value^*^	Coefficient	SE	*P* Value^*^
BCVA (ETDRS score)	Hyperautofluorescent ring	78.9	2.7	<0.0001	75.8	3.4	<0.0001
	Patch pattern^†^	**−17**	**4.1**	**<0.0001**	**−25.2**	**4.9**	**<0.0001**
	Severely decreased FAF^†^	**−38.5**	**4.9**	**<0.0001**	**−42.1**	**5.9**	**<0.0001**
IIIe4 SKVF area (^°2^)	Hyperautofluorescent ring	4936.8	822.9	<0.0001	4772.8	794.3	<0.0001
	Patch pattern^†^	**−2940.5**	**1158**	**0.0125**	−1559.2	930.4	0.0967
	Severely decreased FAF^†^	**−3828**	**1565.9**	**0.0161**	−2414.5	1266.1	0.0592
V4e SKVF area (^°2^)	Hyperautofluorescent ring	7796.7	1031.5	<0.0001	7503.2	1021.5	<0.0001
	Patch pattern^†^	**−4425.5**	**1530.2**	**0.0046**	**−4000.7**	**1430.9**	**0.0061**
	Severely decreased FAF^†^	**−5480.8**	**1823.3**	**0.0032**	**−4331.3**	**1681.9**	**0.0113**
MS (dB)	Hyperautofluorescent ring	13.1	1.6	<0.0001	13.1	2.1	0.0000
	Patch pattern^†^	**−*8.9***	***2.5***	***0.0007***	**−8.5**	**2.8**	**0.0035**
	Severely decreased FAF^†^	**−*12.2***	***2.5***	***<0.0001***	**−11.2**	**2.9**	**0.0003**
CMT (µm)	Hyperautofluorescent ring	298	10.1	<0.0001	299.5	9	<0.0001
	Patch pattern^†^	**−57.8**	**15.5**	**0.0003**	**−83.4**	**14**	**<0.0001**
	Severely decreased FAF^†^	**−102.2**	**18.7**	**<0.0001**	**−112.9**	**15.6**	**<0.0001**
EZ horizontal width (µm)	Hyperautofluorescent ring	3754.3	325.2	<0.0001	3338.3	357.3	<0.0001
	Patch pattern^†^	**−2115.8**	**514.1**	**0.0001**	**−1564.2**	**533.4**	**0.0041**
	Severely decreased FAF^†^	**−3406.3**	**631.6**	**<0.0001**	**−2743.7**	**637.7**	**<0.0001**
EZ vertical width (µm)	Hyperautofluorescent ring	2996.5	337.1	<0.0001	2745	348.8	<0.0001
	Patch pattern^†^	**−1284.1**	**510.4**	**0.0133**	−1021.4	515.3	**0.0500**
	Severely decreased FAF^†^	**−2321.9**	**640.1**	**0.0004**	**−2091.4**	**672.5**	**0.0024**

Patch pattern and severely decreased FAF were associated with significantly worse BCVA, V4e SKVF area, MS, CMT, and EZ horizontal width, compared with hyperautofluorescence ring either in the better- and worse-seeing eyes.

* *P* values are obtained by linear mixed models; statistically significant *P* values are shown in bold.

† Compared with the hyperautofluorescent ring as the refence group.

**Figure.**
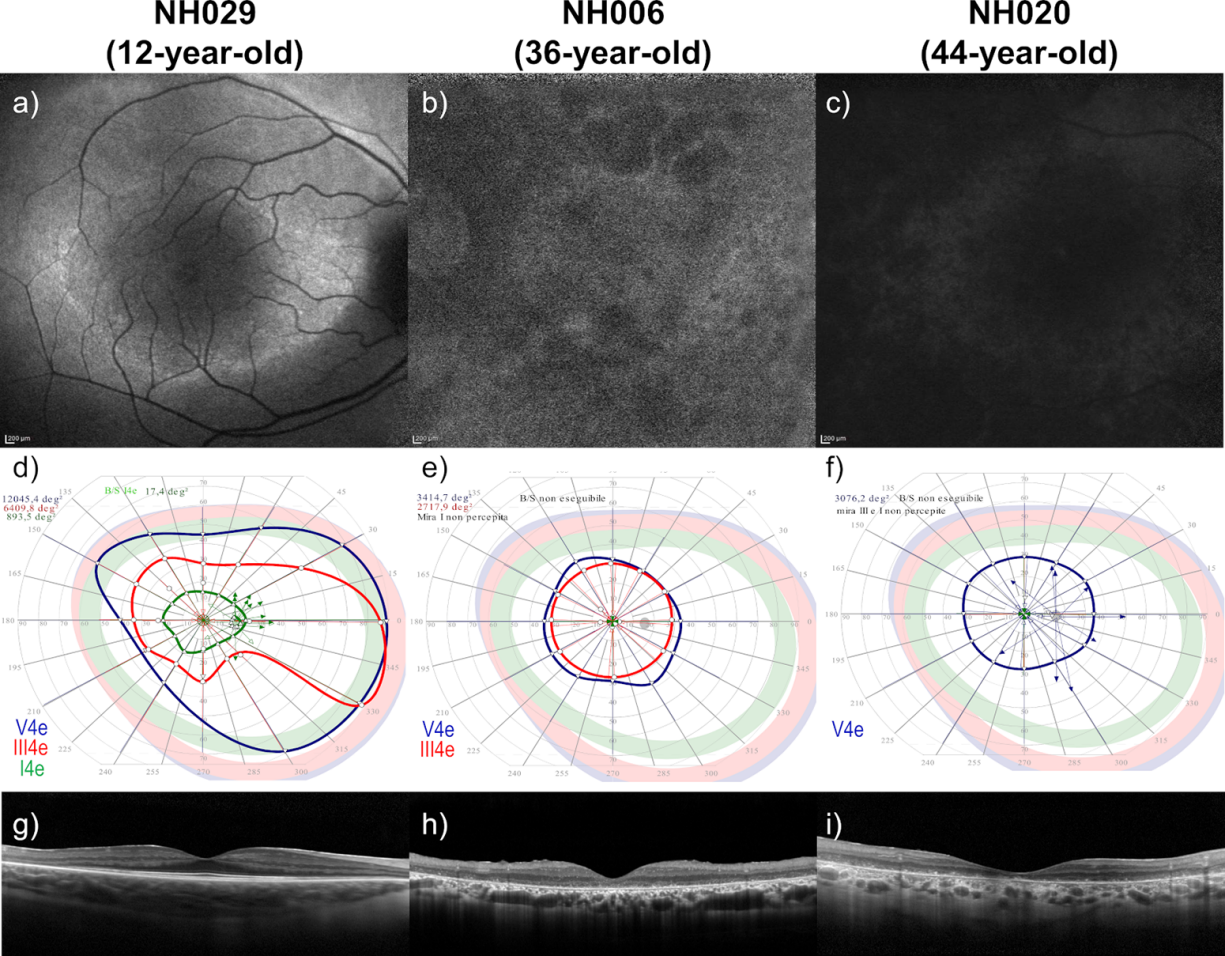
Comparison of SKVF and SD-OCT in three selected patients with different FAF patterns. FAF shows the three most common patterns: hyperautofluorescent ring (**a**), hyperautofluorescent foveal patch (**b**), and abnormal central hypoautofluorescence (**c**). SKVF in the cases with hyperautofluorescent foveal patch (**e**) and with abnormal central hypoautofluorescence (**f**) show a more restricted area compared with the case with hyperautofluorescent ring (**d**). SD-OCT scans show a slightly reduced CMT with preserved EZ band in the case with hyperautofluorescent ring (**g**), markedly decreased CMT with disrupted EZ band in the case with hyperautofluorescent foveal patch (**h**), and markedly reduced CMT without detectable EZ band in the case with abnormal central hypoautofluorescence (**i**).

### Genetic Characterization of the *MYO7A* Cohort

A total of 60 distinct *MYO7A* variants were detected across the 106 alleles of the 53 included patients (Supplementary Table S2, Supplementary Fig. S1). Most variants had already been reported in public repositories such as ClinVar (http://www.ncbi.nlm.nih.gov/clinvar/) and the Leiden Open Variation Database (http://www.lovd.nl/LOVD) or in previous reports,^22^^,^^23^ and only two variants (c.2078del, c.6521del) were novel. In terms of zygosity, more than one-half of the enrolled patients were compound heterozygotes for the identified variants (*n* = 29; 54.7%), whereas the remaining 45% (*n* = 24; 45.3%) were homozygous.

In terms of functional impact, the most frequently encountered variations were missense changes, detected in 57 of the 106 alleles (53.8%). Two missense changes (c.569T>G; p.Leu190Trp and c.1969C>T; p.Arg657Trp) were present in cis on a complex allele and detected at the homozygous state (confirmed by segregation analysis) in patient NH032. The remaining alleles harbored presumably loss-of-function (LoF) variants, namely nonsense variants (*n* = 25 [23.6%]), frameshift variants (*n* = 11 [10.4%]), splice-site variants (*n* = 10 [9.4%]), and a large deletion encompassing exons 43 to 49 (*n* = 2 [1.9%]). Finally, one participant carried a heterozygous in frame indel of three amino acids (*n* = 1 [0.9%]). The most recurrent variant was the missense change c.721C>G (p.Arg241Gly) found in 13.2% of the alleles (*n* = 14); the remaining variants had a lower frequency (found in ≤5 alleles).

More than 94% of the identified variants were classified as pathogenic (*n* = 85/106 [80.2%]) or likely pathogenic (*n* = 15/106 [14.2%]) using the automated implementation of the American College of Medical Genetics and Genomics guidelines in Varsome.^24^ The remaining 5.6% of the alleles (*n* = 6/106) harbored four distinct variants (namely, one missense, two splice site changes, and one synonymous variant predicted to alter a canonical splice site) that were classified as variants of uncertain significance (Supplementary Table S2). These variants of uncertain significance were found in patients who also carried a (likely) pathogenic variant (NH021, NH022, NH024, and NH030), except for the missense variant c.4114G>A (p.Val1372Met), which was detected at homozygous state in patient NH113 (Supplementary Table S2). Moreover, in silico assessment of the synonymous and the two splice site variants using SpliceAI (http://spliceailookup.broadinstitute.org/) strongly supported their possible effect on splicing. Given that the identified genotypes were convincing, and the clinical presentation of the patients was highly specific for causative defects in *MYO7A*, we included the above mentioned five patients harboring variants of uncertain significance in this natural history study.

To seek for possible genotype–phenotype correlations, we stratified the genotypes based on the number of LoF alleles identified in each participant. To this aim, we considered missense changes and in frame indels as nontruncating variants, whereas frameshifts, splicing, and nonsense variants as *bona fide* LoF variants. Therefore, we identified three groups of patients: group A, two nontruncating variants; group B, one LoF and one nontruncating variant; and group C, two LoF variants. As summarized in Table 5, age-adjusted models did not show any significant difference in any of the selected clinical parameters between the three subgroups of patients stratified on the basis of the number of LoF variants. Moreover, there were no significant differences (*P* > 0.40) in the distribution of the three most common macular FAF patterns and the three subgroups of patients stratified on the basis of the number of LoF variants.

**Table 5.** Age-adjusted Models Comparing Patients Stratified According to the Number of LoF Variants

		Better-Seeing Eyes			Worse-Seeing Eyes		
Parameter	Factor or Variable	Coefficient	SE	*P* Value^*^	Coefficient	SE	*P* Value^*^
BCVA (ETDRS score)	Group A	79.1	5.9	<0.0001	77.4	7.0	<0.0001
	Group B vs. group A	1.2	5.6	0.8312	2.1	6.7	0.7542
	Group C vs. group B	6.1	5.7	0.2898	9.2	6.7	0.1779
	Age adjustment^†^	−0.4	0.1	0.0029	−0.6	0.2	0.0007
III4e SKVF area (^°2^)	Group A	8645.8	1369.2	<0.0001	10506.1	1602.5	<0.0001
	Group B vs. group A	−2015.0	1317.5	0.1344	−1621.8	1359.7	0.2408
	Group C vs. group B	−1488.7	1339.0	0.2732	−1862.0	1507.3	0.2247
	Age adjustment^†^	−131.3	34.3	0.0005	−180.9	43.6	0.0002
V4e SKVF area (^°2^)	Group A	13555.5	1591.6	<0.0001	13840.4	1745.1	<0.0001
	Group B vs. group A	−2868.2	1462.8	0.0566	−1210.5	1419.2	0.3988
	Group C vs. group B	−2296.7	1519.5	0.1381	−2252.0	1639.5	0.1772
	Age adjustment^†^	−195.2	39.3	<0.0001	−238.4	46.2	<0.0001
MS (dB)	Group A	269.1	22.5	<0.0001	276.9	24.9	<0.0001
	Group B vs. group A	8.4	21.4	0.6953	12.7	23.0	0.5845
	Group C vs. group B	20.2	21.9	0.3616	17.3	23.4	0.4636
	Age adjustment^†^	−0.5	0.5	0.3257	−1.1	0.6	0.0982
CMT (µm)	Group A	24.6	3.9	<0.0001	22.2	4.5	<0.0001
	Group B vs. group A	−3.3	3.4	0.3395	−1.7	3.9	0.6688
	Group C vs. group B	−4.2	3.9	0.2881	−3.9	4.4	0.3751
	Age adjustment^†^	−0.4	0.1	0.0013	−0.4	0.1	0.0093
EZ horizontal width (µm)	Group A	4565.4	869.9	<0.0001	4278.0	904.0	<0.0001
	Group B vs. group A	−174.3	857.5	0.84	−632.3	850.4	0.4615
	Group C vs. group B	−203.3	843.9	0.8109	−508.4	862.3	0.5587
	Age-adjustment^†^	−51.7	21.7	0.0218	−41.1	23.1	0.0831
EZ vertical width (µm)	Group A	3956.8	858.9	<0.0001	4236.7	914.8	<0.0001
	Group B vs. group A	−869.5	809.7	0.2893	−1005.7	855.8	0.2473
	Group C vs. group B	−1264.6	846.3	0.1429	−1155.7	854.8	0.1844
	Age adjustment^†^	−25.8	21.1	0.2276	−34.0	22.4	0.1369

Group A includes those with two nontruncating variants, group B includes those with one LoF and one nontruncating variant, and group C includes those with two LoF variants.

* *P* values are obtained by mean response profiles models.

† The models are adjusted for the age of the patients at the baseline visit.

## Discussion

We report the results of a multicentric longitudinal study designed to assess the disease course in a large cohort of European patients (*n* = 50) with USH1B, carrying biallelic pathogenic variants in *MYO7A* and describe for the first time the progression of the disease in a prospective longitudinal setting. In particular, we found a relatively slow disease progression with a statistically significant worsening only of BCVA over a 2-year follow-up in the overall cohort, and a statistically significant reduction of EZ band in the subgroup of patients aged 21 to 40 years.

The natural history of the retinal degeneration in *MYO7A* patients has already been investigated in a few recent cross-sectional^10^ and retrospective studies.^5^^,^^25^^,^^26^ Overall, the data from our cohort confirm the early disease onset with significant retinal abnormalities and visual impairment also in the youngest cases.

In particular, the observed decline of BCVA by 2-ETDRS letters over a 2-year follow-up was consistent with the findings reported in literature (e.g., around 1 ETDRS letter per year reported by Khateb et al.^5^ and 1.85%/year described by Lenassi et al.^25^). In contrast, we did not observe a significant decrease in the EZ band width, reported in the retrospective study by Khateb et al.^5^

Visual field results could not be compared with those reported in previous reports owing to different testing modalities. All published studies performed manual Goldmann kinetic visual field tests,^5^^,^^25^^,^^26^ although we preferred a semiautomatic evaluation with a computerized perimeter to standardize measurements in multicentric study context.

Further, we observed that the baseline values of BCVA, SKVF area, MS, and EZ band were strongly age related, confirming slow disease progression with relatively preserved macular function and morphology within the first three decades of life.

In addition, our findings confirmed that the FAF patterns (i.e., hyperautofluorescent ring, hyperautofluorescent foveal patch, and abnormal central hypoautofluorescence) characterize different morphological stages of retinal impairment. This finding was reported previously in other cohorts of USH patients, highlighting a progressive spatial correlation among FAF worsening, outer retina disruption, and MS reduction.^17^^,^^27^^,^^28^

In our cohort of *MYO7A* patients, the hyperautofluorescent ring, as well as normal FAF, corresponded with the earliest stage of retinal impairment and was associated with a relatively preserved visual function in terms of BCVA (i.e., >70 ETDRS score), SKVF (area V4e >10,000^°2^), and MS (e.g., >10 dB). The abnormal central hypoautofluorescence, instead, was consistent with an advanced disease stage and significantly related to poor visual function in terms of BCVA (i.e., <50 ETDRS score), SKVF (area V4e <5000^°2^), and MS (e.g., <5 dB). Furthermore, the FAF patterns correlated with the morphological alteration of the outer retina observed by SD-OCT. In particular, a preserved EZ band (i.e., > 2000 µm) was associated with the presence of a hyperautofluorescent ring, whereas an abnormal central hypoautofluorescence was associated with a minimal residual EZ band (i.e., <1000 µm).

These morphological findings are particularly relevant for patients with USH, because the association of deafness and low vision or blindness prevents accurate completion of psychophysical tests. In this regard, our results could be useful to define appropriate inclusion criteria and suitable windows of treatment for gene therapy clinical trials. In addition, considering the robust real-life experience on the safety and efficacy of voretigene neparvovec treatment for biallelic *RPE65*-retinopathy, which offered insights on the unexpected occurrence of chorioretinal atrophy in treated patients, it seems to be of utmost importance to carefully select the patients who will be included in a future phase 1 clinical trial on the basis of FAF patterns.^29^^–^^33^ For example, patients with abnormal central hypoautofluorescence, which was indicative of RPE atrophy in our cohort, would represent the least appropriate candidates for gene therapy, even when retinal thickness is preserved, because any treatment benefit (or potential toxic effect) would be difficult to identify by current clinical biomarkers (e.g., SD-OCT or EZ band width, CMT, visual acuity, visual field). Instead, the hyperautofluorescent foveal patch pattern, which in our cohort represented an intermediate stage of the disease and progressed only in few cases (10%) to abnormal central hypoautofluorescence over the 2-year follow-up, would seem to be more suitable to reveal both the potential benefits and eventual toxic adverse effects of an experimental treatment.

The current study has some limits, mainly related to the study design realized in the framework of a European Project (grant 754848 - “Ushther”) aimed to support clinical development of an innovative gene therapy approach. First, the inclusion and exclusion criteria have been designed to focus on patients suitable for a gene therapy approach, and thus excluding cases with USH-related complications (e.g., severe CML, vitreoretinal pathology), which represent a relevant part of the disease spectrum. Second, the follow-up period is relatively short to show a significant progression; however, a time-frame of 2 years was chosen to identify potential biomarkers that could be useful in a short-term evaluation of effects of an experimental treatment.

In conclusion, our multicentric longitudinal study confirmed, for the first time in a prospective setting, the relatively slow natural course of the disease*,* which complicates the identification of reliable and reproducible outcomes to adopt in gene therapy trials. Furthermore, it provided valuable information about FAF as an objective readout to accurately monitor and stage the progression of RP in patients with USH1B. This point is particularly crucial for an efficient planning of future therapeutic trials, especially now that approaches to tackle the large gene size of *MYO7A* are under development.^12^^,^^14^

## Supplementary Material

Supplement 1

Supplement 2

Supplement 3
