# Supplementary material for: Multicentric Longitudinal Prospective Study in a European Cohort of MYO7A Patients: Disease Course and Implications for Gene Therapy
Source: Invest Ophthalmol Vis Sci. 2024 Jun 17;65(6):25. doi: 10.1167/iovs.65.6.25 (PMC11185270; doi:10.1167/iovs.65.6.25)
Supplement: Supplement 1 [file iovs-65-6-25_s001.pdf]

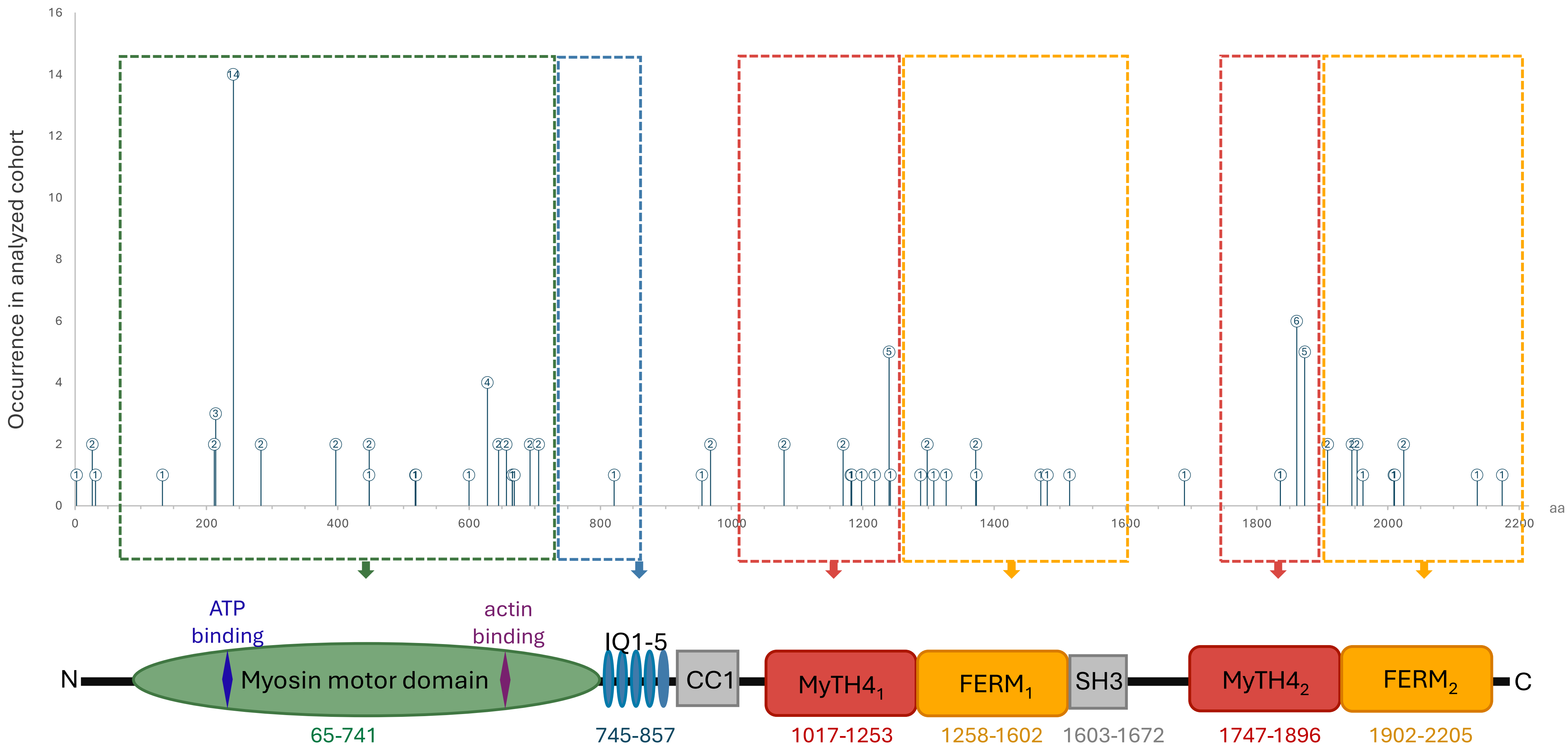

Supplementary Figure 1 Variant distribution along the MYO7A protein structure.
(upper panel) Lollipop plot showing the position of the identified variants along the MYO7A protein (x axis; numbers indicate amino acid position) and their frequency (y axis; values indicate number of pathogenic alleles) in the study cohort. Dotted frames indicate the MYO7A functional domains corresponding to these positions.
(lower panel) Schematic drawing of the MYO7A functional protein domains (according to http://www.uniprot.org/uniprotkb/B9A012/entry). The amino acid range corresponding to each functional domain is indicated below (not to scale).
Abbreviations: aa, amino acid; CC1, coiled-coil region; FERM, band 4.1-ezrin-radixin-moesin; IQ, isoleucine-glutamine motif; MyTH4, Myosin Tail Homology 4; SH3, Src homology 3.
